# Antibody correlates of risk of clinical malaria in an area of low and unstable malaria transmission in western Kenya

**DOI:** 10.1186/s12936-025-05300-1

**Published:** 2025-03-04

**Authors:** Eliud O. Odhiambo, Kagan A. Mellencamp, Bartholomew N. Ondigo, Karen E. S. Hamre, James G. Beeson, D. Herbert Opi, David L. Narum, George Ayodo, Chandy C. John

**Affiliations:** 1 Department of Microbiology and Immunology, Indiana University School of Medicine, Indianapolis, USA; 2 Ryan White Center for Pediatric Infectious Diseases & Global Health, Indiana University School of Medicine, Indianapolis, USA; 3 Department of Biochemistry and Molecular Biology, Egerton University, Nakuru, Kenya; 4 The Carter Center, Atlanta, USA; 5 Burnet Institute, Melbourne, Australia; 6 National Institutes of Health (NIAID/NIH), National Institute of Allergy and Infectious Diseases, Maryland, USA; 7 Jaramogi Oginga Odinga University of Science and Technology, Bondo, Kenya

**Keywords:** *Plasmodium falciparum*, Antibodies, Nested case–control, Low and unstable malaria transmission, Highland, Clinical malaria, Risk

## Abstract

**Background:**

Defining antibody correlates of protection against clinical malaria in areas of low and unstable transmission is challenging because of limited malaria cases in these areas. Additionally, clinical malaria affects both adults and children in areas of low and unstable transmission, but it is unclear whether antibody correlates of protection against malaria differ with age.

**Methods:**

Blood samples were obtained from 5753 individuals in Kenyan highland area with low and seasonal malaria transmission in 2007 and recorded episodes of clinical malaria in this population from 2007 to 2017. Using a nested case–control study design, participants who developed clinical malaria (cases) were matched by age and village to those who did not (controls). Immunoglobulin (Ig)G, IgG1, IgG3, IgA and IgM responses to 16 *Plasmodium falciparum* antigens were compared in individuals < 5 years old (80 cases vs. 240 controls), 5–14 years old (103 cases vs. 309 controls) and ≥ 15 years old (118 cases vs. 354 controls). Antibody level was correlated with risk of clinical malaria, adjusted for malaria exposure markers.

**Results:**

In all age groups, most antibodies were not associated with risk of clinical malaria. In children < 5 years, higher levels of IgG to GLURP-R2 and MSP-2, IgG1 to GLURP-R2, and IgG3 to MSP-2 were associated with reduced risk of clinical malaria, while higher IgG3 levels to CSP were associated with increased risk of clinical malaria. In children 5–14 years and individuals ≥ 15 years, higher antibody levels to multiple *P. falciparum* antigens were associated with an increased risk of clinical malaria, and none were associated with decreased risk of clinical malaria.

**Conclusions:**

Antibody correlates of protection against clinical malaria were observed only in children < 5 years old in this area of low and unstable malaria transmission. In older children and adults in this area, some antibody responses correlated with increased risk of clinical malaria. Future studies in low malaria transmission areas should evaluate the comparative contributions of cellular and humoral immunity to protection from clinical malaria in young children versus older children and adults.

**Supplementary Information:**

The online version contains supplementary material available at 10.1186/s12936-025-05300-1.

## Background

Every year, malaria infects > 200 million individuals and causes > 600,000 deaths [1]. Most malaria cases occur in sub-Saharan Africa, where > 95% are due to *Plasmodium falciparum* [1, 2]. Malaria transmission varies among affected regions, with areas of low, moderate and high rates of malaria transmission [3, 4]. Protection against clinical malaria often develops with age and repeated exposure in settings of high malaria transmission [5, 6]. In such settings, adults tend to experience asymptomatic malaria due to partial immunity to clinical malaria after multiple malaria exposures, whereas children < 5 years are more likely to exhibit clinical symptoms with parasitaemia [7, 8].

Numerous studies have reported immune correlates of protection against clinical malaria in areas of high malaria transmission, including antibodies to multiple pre-erythrocytic [9–12] and blood-stage [13–20] malaria antigens. Most studies have reported on IgG antibody correlates of protection against clinical malaria [12, 19, 20], but some have reported associations of IgG subclass antibodies, notably IgG1 and IgG3 [13–17]. In recent years, studies have also shown associations of IgA [21] and IgM [22, 23] antibodies to specific *P. falciparum* antigens with protection from clinical malaria. However, few studies of immune correlates of protection have been conducted in areas of low and unstable malaria transmission. Such studies are challenging because the low incidence of clinical malaria in these sites typically results in sample sizes insufficient for evaluating protection from clinical malaria. In these areas, adults are often at risk of clinical malaria, in some areas at a similar level to children [24] and in others at a lower level than children but still with substantial risk [25]. Evaluation of immune correlates of protection in areas of low transmission, and whether they differ with age, is important to understanding how to design and evaluate interventions to reduce malaria, particularly vaccines, for individuals living in these areas.

Prior research in this highland area of Kenya with low and unstable malaria transmission over a 6 year follow-up period showed that IgG antibodies to CSP, LSA-1, and GLURP-R2, out of 11 antigens tested, were associated with protection from clinical malaria. The present study expanded on the prior study by evaluating association of antibodies in this cohort with risk of clinical malaria over 10 year follow-up in three age groups (children < 5 years of age, children 5–14 years of age, and individuals ≥ 15 years of age) and with testing of not only IgG antibodies, but IgG1, IgG3, IgM and IgA antibodies to 16 vaccine-candidate antigens from three stages of malaria parasite life cycle in human host including Pfs230, a sexual stage antigen.

## Methods

### Study area, population, and design

A cohort study of clinical malaria surveillance was conducted in the Kipsamoite and Kapsisiywa regions of Nandi County, highland areas of low and unstable transmission in western Kenya [26, 27] from 2007 to 2017. Households that consented to the study were included in demographic surveys and passive surveillance of clinical malaria at the local health centers [27, 28]. In 2007, 5753 out of approximately 8000 people living in the region agreed to participate in a site-wide blood collection for study testing. One blood sample was obtained from each study participant between April and June 2007. To diagnose asymptomatic malaria in all collected samples, blood smears were prepared and examined. Following blood sample collection, field assistants (FA) conducted biannual (2007–2008) and annual (2009–2017) demographic surveys, gathering data on markers of malaria exposure such as elevation and distances to closest forest, swamp, and health clinic using GPS, roof material, indoor residual spraying, insecticide-treated bed net use and travel outside of the study area.

A nested case–control design within the cohort study was employed to evaluate antibody correlates of risk of clinical malaria. Participants from the initial site-wide collection who developed clinical malaria during subsequent malaria surveillance from June 2007 to May 2017, when the region experienced a malaria outbreak, were included as cases, while individuals who did not develop malaria within the same time period as cases were termed controls. In this population, the occurrence of asymptomatic malaria (presence of *P. falciparum* parasitaemia in individuals with no symptoms of malaria) was extremely rare, with only 14 positive blood smear results found among 5753 samples collected, representing < 0.3% of the total. Consequently, in the current study, only participants that tested negative on smear examination at the time of blood collection were included. Furthermore, repeated malaria episodes were rare in this community: only 19 of 301 cases (6.3%) had repeat episodes, with a maximum of 2 episodes per person over the 10 years of follow-up. Clinical malaria was defined as a positive blood smear for *P. falciparum* at any density in combination with measured fever (axillary temperature ≥ 37.5 °C) or history of fever or headache, clinical criteria that were previously shown to be sensitive and specific for malaria in this study area [29]. Prior studies have also shown that in this area, fever could be attributed to malaria even at the lowest detectable levels by microscopy because asymptomatic parasitaemia is rare in this community [29]. Three controls per case were matched according to age at blood collection and village. For cases aged < 64 years, controls were matched within 2 years of age. For cases aged ≥ 65 years, controls were matched within 5 years of age given the limited number of older participants in this age range. Matched case–control groups were categorized into three age groups for analyses: children < 5 years at enrolment, and individuals 5–14 years and ≥ 15 years at enrolment. The stratification by these age groups was chosen because clinical malaria in areas of high malaria transmission commonly affects children < 5 years mainly due to their immature immunity [1], but children 5–14 years were reported to have the highest incidence of clinical malaria in this study population from 2007 to 2013, as previously published [28]. Cases and matched controls (80 cases vs. 240 controls of children < 5 years, 103 cases vs. 309 controls of children 5–14 years, and 118 cases vs. 354 controls of individuals ≥ 15 years) were evaluated.

### Human subjects protection and ethical review

Written informed consent was obtained from household heads when recruited for participation in the demographic and malaria surveillance, from participants during the site-wide blood collection and when visiting the clinic with malaria symptoms. This study was approved by the Kenya Medical Research Institute Ethical Review Committee and the Indiana University Institutional Review Board.

### Recombinant antigens

The three stages of the *P. falciparum* life cycle (pre-erythrocytic, erythrocytic, and sexual) were represented by the 16 recombinant antigens used for antibody testing. Fifteen vaccine candidate antigens were chosen based on data showing association of antibodies to the antigens with protection against clinical malaria [13, 20, 28, 30–37], and Pfs230, a sexual stage antigen, was used as a comparison antigen that generates antibodies which should not be associated with protection against clinical malaria [38–40]. Pre-erythrocytic antigens included the full-length circumsporozoite protein (CSP); 3D7 strain liver-stage antigen 1 (LSA-1); and thrombospondin-related adhesion protein (TRAP). The erythrocytic-stage antigens comprised the 3D7 strain apical membrane antigen 1 (AMA-1); erythrocyte binding antigen 140 region III-V (EBA-140 RIII-V); erythrocyte binding antigen 175 (EBA-175) regions II (RII) and III-V (III-V); N-terminal non-repetitive region (R0) and C-terminal repetitive region (R2) of glutamate-rich protein (GLURP); 3D7 strain merozoite surface protein 1 (MSP-1_42_), 2 (MSP-2) and 3 (MSP-3); and the reticulocyte binding protein homologue 2 (Rh2.2030), 4.2 (Rh4.2) and 5.1 (Rh5.1) [41]. Lastly, the sexual stage antigen was Domain 1 of Pfs230 [42]. Full-length CSP, a.a 19–424, was obtained from Creative Diagnostics (Cat # DAG1344F). Recombinant proteins MSP-2, EBA-140 RIII-V, EBA-175 RIII-V, Rh2.2030, Rh4.2, and Pfs230 [16, 43–45] were provided by James Beeson, Burnet Institute, Melbourne, Australia; recombinant Rh5.1 [41] by Simon Draper, University of Oxford, England, UK; recombinant EBA-175, MSP-1_42,_ and MSP-3 by David Narum, National Institute of Allergy and Infectious Diseases; and recombinant GLURP-R0 and GLURP-R2 antigens by Michael Theisen of the Statens Serum Institut, Copenhagen, Denmark.

### Antigen coupling and antibody testing

Antigen coupling to MagPlex microspheres (Luminex Corp., TX) was performed as previously described [46]. A Luminex bead assay was used to test IgG, IgG1, IgG3, IgM, and IgA antibodies against 16 recombinant antigens, as previously described [46–48]. Modification to the previously established method included plasma dilutions. Plasma samples were diluted 1:100 for IgG1 and IgG3, 1:200 for IgA, and 1:500 for IgG and IgM. Each plate included a positive control sample, a pool of plasma samples from Kenyans living in malaria-endemic areas that are known to be seropositive for malaria [49], blanks containing phosphate-buffered saline diluents, and nine plasma samples from North American controls (NACs) who had never been exposed to malaria. The cases and their matched controls were organized side by side in the same plate. Controls and blanks were included to assess consistency of testing across the plates. The results from MagPix were presented as the median fluorescence intensity (MFI). MFI values for positive controls across plates were highly correlated (Spearman’s rho range 0.932–1.000, p < 0.0001 for all comparisons) and varied minimally across plates (mean (SD) coefficient of variation for positive control values across plates was 8.80% (3.86%)).

### Statistical analysis

Antibody levels were expressed in arbitrary units (AU) by dividing the MFI of the test sample by the sum of the mean MFI plus three standard deviations (SD) of the NACs. Both continuous antibody levels (AU values log-transformed to base 10) and dichotomized antibody responses (AU ≥ 1 indicating a positive response) were considered in the analyses. Poisson regression was used to estimate differences in the overall clinical malaria incidence per 1000 person-years from 2007 to 2017 across the three age groups: < 5 years, 5–14 years and ≥ 15 years. The proportion of participants with positive antibody responses for each antigen across the age groups was compared using Chi-squared tests. All other statistical analyses were performed using conditional logistic regression to account for age and village matching in the study design. The balance of age between the cases and controls after matching to rule out potential residual confounding by age was also evaluated. Odds ratios (ORs) were estimated using conditional logistic regression to ascertain the associations between antibody responses to the tested antigens and the development of clinical malaria over follow-up. As previously described [28], a purposeful selection of covariates method was used to determine the final adjusted models and conducted sensitivity analyses that excluded data for matched groups that were potentially poorly fit or overly influential. Final models were adjusted for sex and five surrogate markers of malaria [28] household elevation, distance to the closest forest, roof material, indoor residual spraying, and personal use of insecticide-treated bed nets. Furthermore, the predictive capability of single versus combined antibody responses for clinical malaria development, as demonstrated in previous studies [28, 50–52], was evaluated using receiver operating characteristic (ROC) analysis on antibody responses significantly associated with malaria risk. All statistical analyses were performed using Stata/SE v18.0 (Stata Corp., College Station, TX, USA). P values < 0.05 were considered statistically significant.

## Results

### Malaria incidence according to age group

**Fig. 1:**
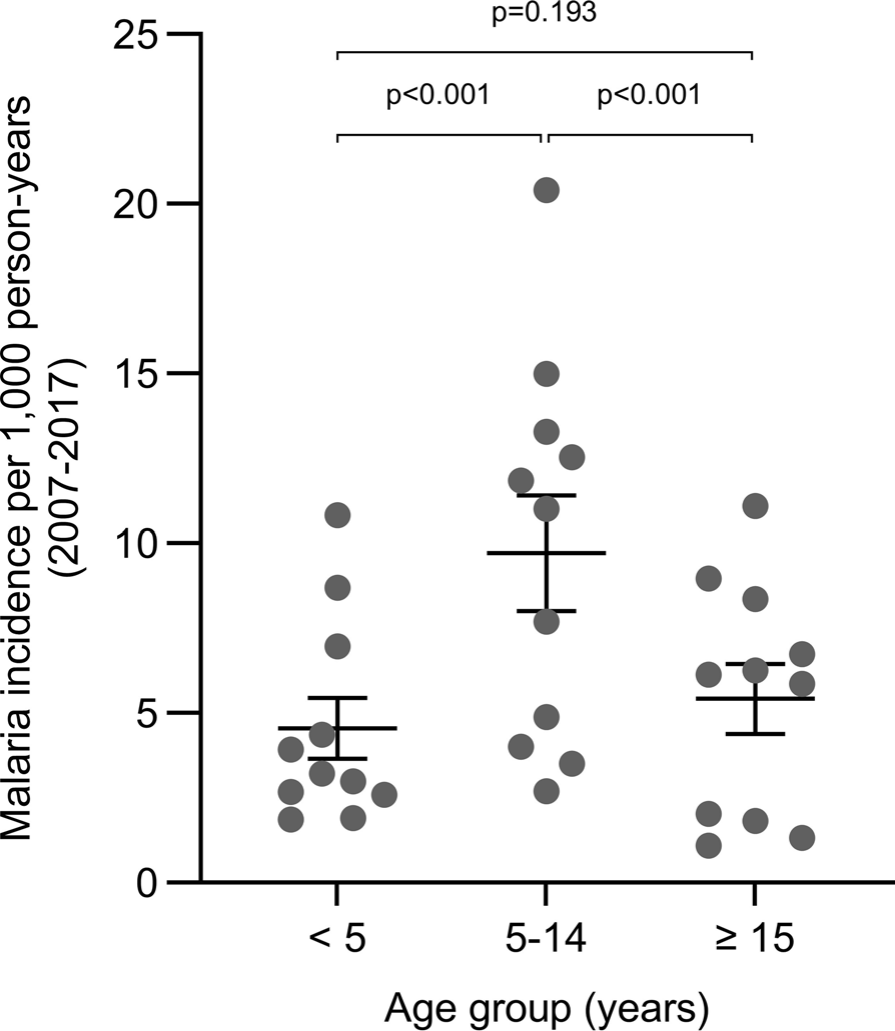
Overall clinical malaria incidence per 1000 person-years in Kipsamoite and Kapsisiywa, western Kenya, 2007–2017, by age group. Dots represent yearly clinical malaria incidence from 2007 to 2017. Confidence intervals (error bars) and differences between groups estimated using Poisson regression

Children 5–14 years had a higher malaria incidence (mean (95% confidence interval (CI)) 9.71 (8.58, 10.96) cases per 1000 person-years), compared to children < 5 years 4.65 (3.55, 5.97), p < 0.001) and individuals ≥ 15 years 5.56 (4.98, 6.21), p < 0.001), but incidence was similar in the children < 5 years and individuals ≥ 15 years (p = 0.193, Fig. 1). Trends in malaria incidence by age group were consistent over time from 2007 to 2017 (Fig. S1). Ages were similar between cases and controls in each age group (Table S1, p > 0.05 for all), demonstrating a balanced age distribution after matching. Most cases occurred within the second 5 years of follow-up (2008–2012, 108 cases [35.9%]; 2013–2017, 193 cases [64.1%]).

### Proportion of individuals who were antibody positive to *P*. *falciparum* antigens, by age group

**Fig. 2:**
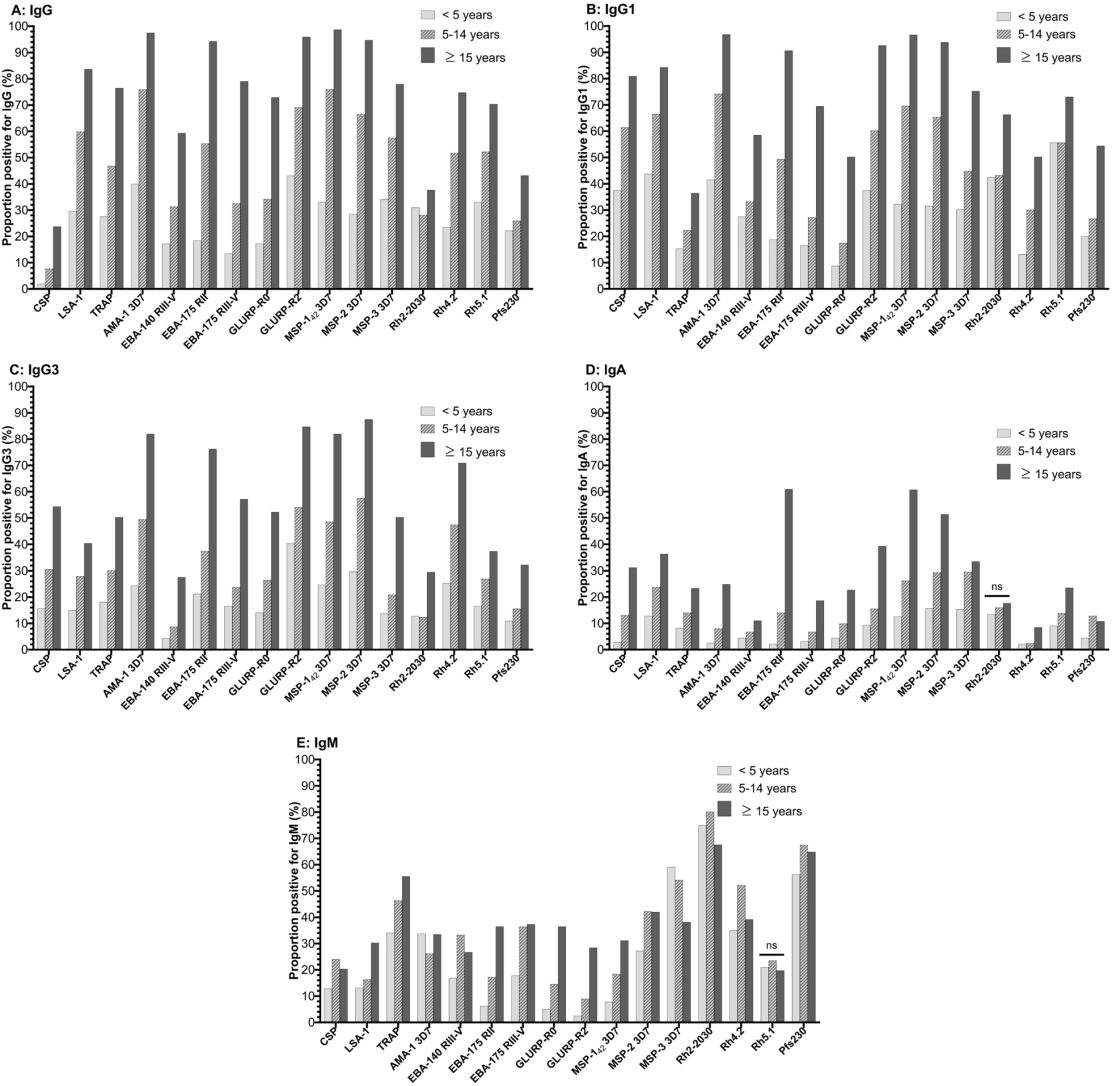
Bar charts depicting percentage of participants positive for **A** IgG, **B** IgG1, **C** IgG3, **D** IgA and **E** IgM to multiple *P. falciparum* antigens according to age group. Individuals were considered positive for an antibody response if the antibody level in arbitrary units was ≥ 1. Differences between age groups estimated using Pearson’s Chi-squared tests and were significant at p < 0.05 unless noted as nonsignificant (ns)

The proportion of participants with IgG, IgG1, IgG3, and IgA antibodies to *P. falciparum*) antigens increased with age, except for the proportion of IgA antibodies to Rh2-2030, which did not differ by age (Fig. 2A–D). The proportion of participants with IgM antibodies to *P. falciparum* antigens also increased with age, except for IgM antibodies to Rh5.1, which did not differ by age, and IgM antibodies to MSP-3 and Rh2-2030, which decreased with age (Fig. 2E).

### Antibody levels and association with risk of clinical malaria

**Fig. 3:**
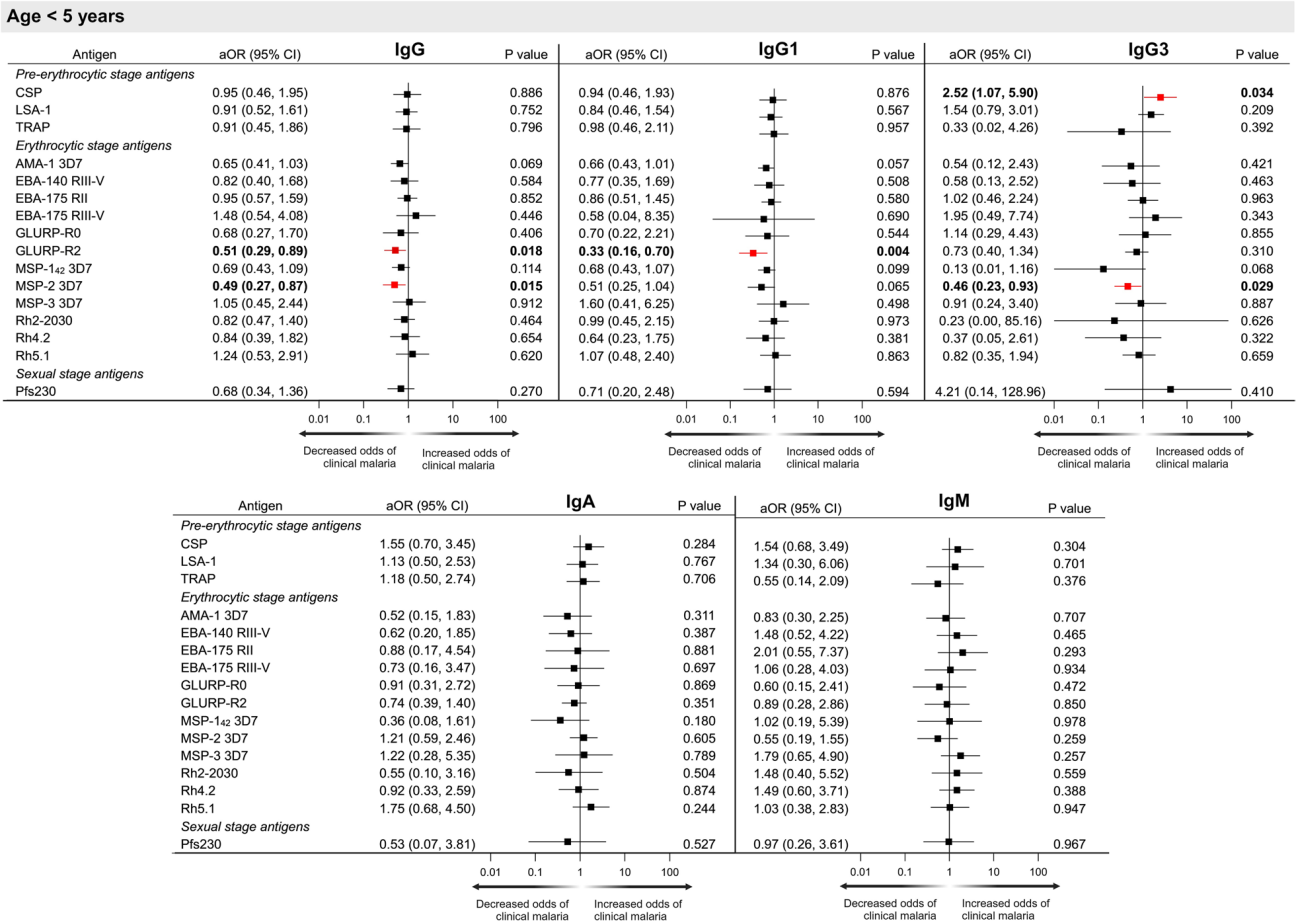
Forest plots representing the association between IgG, IgG1, IgG3, IgA and IgM antibody levels (arbitrary units) to multiple *P. falciparum* antigens and risk of clinical malaria in children < 5 years. ^a^Adjusted odds ratios (aOR) and p values obtained using conditional logistic regression adjusted for bed net use, household treatment by indoor residual spraying, roof material, distance to nearest forest and elevation. Significant aORs at p < 0.05 are depicted in red

**Fig. 4:**
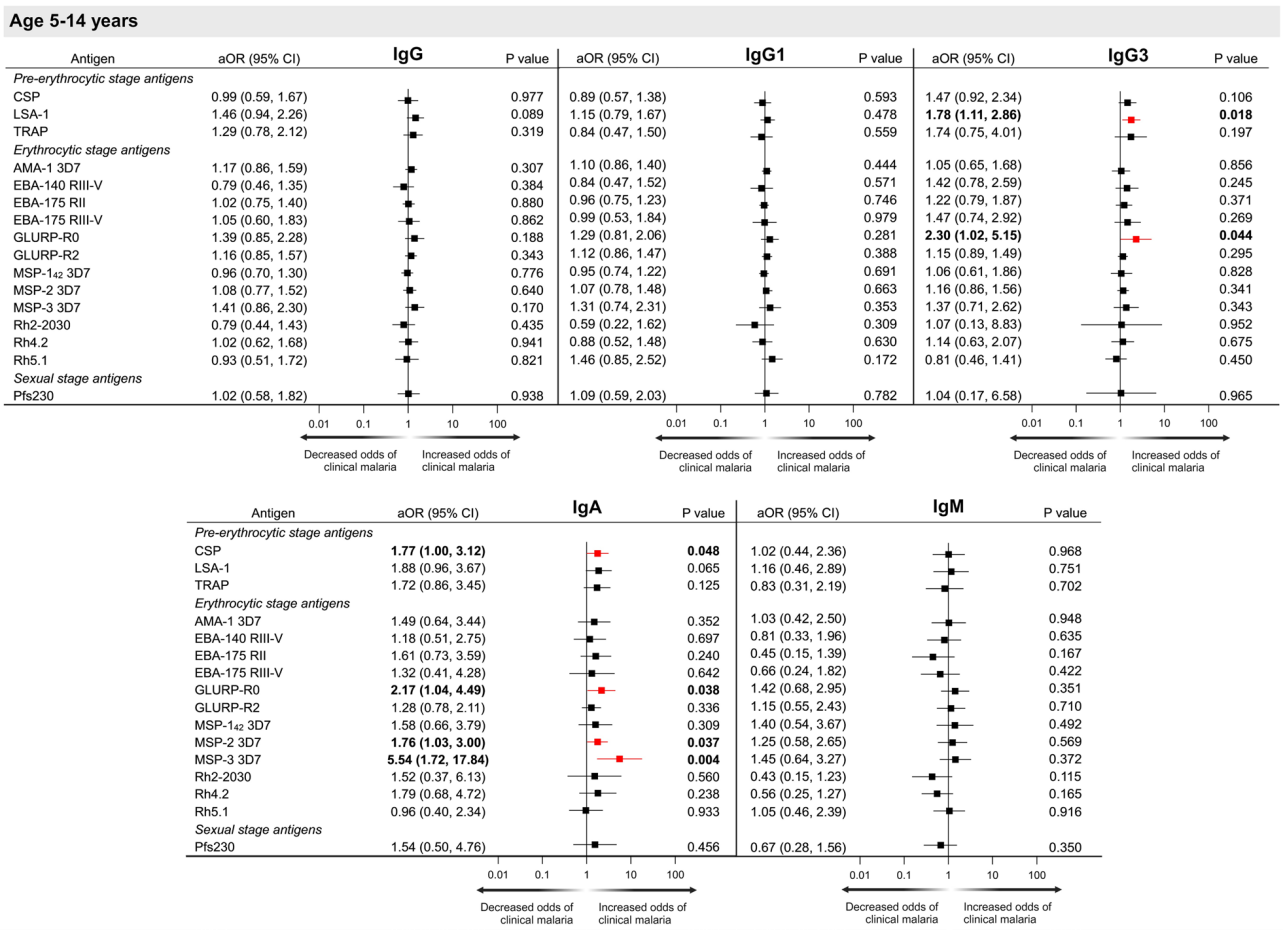
Forest plots representing the association between IgG, IgG1, IgG3, IgA and IgM antibody levels (arbitrary units) to multiple *P. falciparum* antigens and risk of clinical malaria in children 5–14 years. ^a^Adjusted odds ratios (aOR) and p values obtained using conditional logistic regression adjusted for bed net use, household treatment by indoor residual spraying, roof material, distance to nearest forest and elevation. Significant aORs at p < 0.05 are depicted in red

**Fig. 5:**
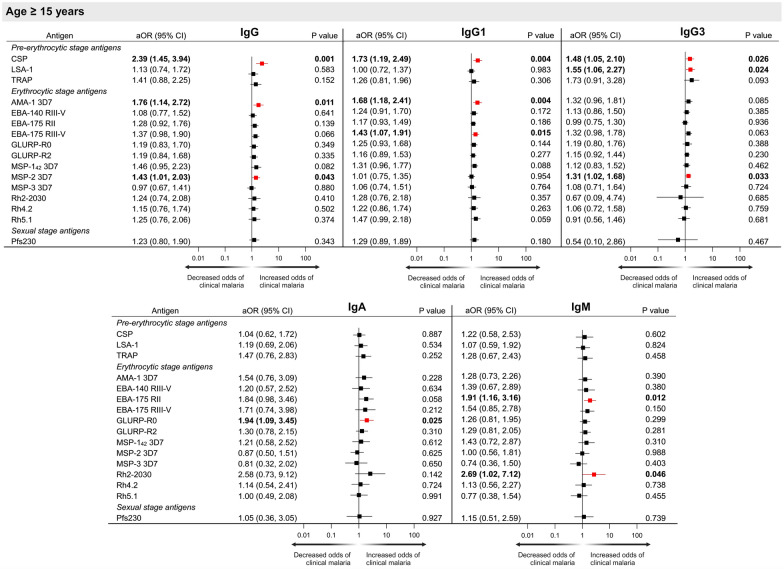
Forest plots representing the association between IgG, IgG1, IgG3, IgA and IgM antibody levels (arbitrary units) to multiple *P. falciparum* antigens and risk of clinical malaria in individuals ≥ 15 years. ^a^Adjusted odds ratios (aOR) and p values obtained using conditional logistic regression adjusted for bed net use, household treatment by indoor residual spraying, roof material, distance to nearest forest and elevation. Significant aORs at p < 0.05 are depicted in red

Next, associations between antibodies to multiple *P. falciparum* antigens and the development of clinical malaria over 10-year follow-up were examined. Because risk of clinical malaria differed significantly according to age, evaluation of antibodies and risk of clinical malaria was done according to age group (< 5 years, 5–14 years, ≥ 15 years of age). Primary analysis was a comparison of antibody levels to risk of clinical malaria (Figs. 3, 4, 5). Antibody levels in AUs were log-transformed to base 10, so adjusted odds ratios (aOR) reflect the odds of developing clinical malaria for every tenfold increase in AU after adjustment for sex and multiple factors associated with malaria exposure in this region (household elevation, distance to closest forest, type of roofing material, indoor residual spraying, and personal use of insecticide-treated bed nets).

The relationship of presence of antibodies (yes/no) was also assessed in secondary analysis (Figs S2–S4). Results were similar to those when assessing antibody levels, but stronger associations were found with the use of antibody levels, which were primary predictor variables for this study.

### Antibody levels and association with risk of clinical malaria in children < 5 years of age

In all age groups, most antibodies to *P. falciparum* antigens were not associated with risk of clinical malaria. However, in children < 5 years of age, increasing levels of IgG and IgG1 antibodies against GLURP-R2 (adjusted odds ratio (aOR) [95% confidence interval (CI)], 0.51 [0.29, 0.89] and 0.33 [0.16, 0.70], respectively), and IgG and IgG3 against MSP-2 (0.49 [0.27, 0.87] and 0.46 [0.23, 0.93], respectively), were associated with a decreased risk of clinical malaria (Fig. 3). In contrast, IgG3 to CSP was associated with an increased risk of clinical malaria (2.52 [1.07, 5.90]), while IgA and IgM antibodies against *P. falciparum* antigens were not associated with the risk of clinical malaria (Fig. 3).

### Antibody levels and association with risk of clinical malaria in children 5–14 years of age

In children 5–14 years of age at enrolment, increasing antibody levels to specific *P. falciparum* antigens were associated with an increased risk of developing clinical malaria (Fig. 4), including IgG3 to LSA-1 and GLURP-R0 (1.78 [1.11, 2.86] and 2.30 [1.02, 5.15], respectively), and IgA to CSP, GLURP-R0, MSP-2 and MSP-3 (1.77 [1.00, 3.12], 2.17 [1.04, 4.49], 1.76 [1.03, 3.00], and 5.54 [1.72, 17.84], respectively). No association with the development of clinical malaria was observed for IgG, IgG1, or IgM antibodies to any *P. falciparum* antigen (Fig. 4).

### Antibody levels and association with risk of clinical malaria in individuals ≥ 15 years of age

In individuals ≥ 15 years of age at enrolment, increasing antibody levels to numerous *P. falciparum* antigens were associated with a greater risk of developing clinical malaria (Fig. 5), including IgG to CSP, AMA-1, and MSP-2; IgG1 to CSP, AMA-1, EBA-175 RIII-V; IgG3 to CSP, LSA-1, and MSP-2; IgA to GLURP-R0; and IgM to EBA-175 RII and Rh2-2030 (Fig. 5).

### Antibodies and association with risk of clinical malaria in years 1–5 vs. 6–10 of follow-up

In all 3 age groups in the first 5 years vs. years 6–10 of follow-up, antibodies and association with risk of clinical malaria were assessed to determine if associations were stronger in one period vs. the other, since the episodes in the first 5 years were closer to the time when plasma samples for antibodies were obtained. In children < 5 years of age at enrolment, trends towards decreased risk of malaria were seen for most antibodies in years 1–5, but not years 6–10 (Table S2). In individuals 5–14 years and ≥ 15 years, all significant associations (p < 0.05) at both time points were for increased risk of clinical malaria, except for IgM antibodies to some antigens in individuals 5–14 years of age in years 1–5 of follow-up, which were significantly associated with a decreased risk of clinical malaria (Tables S3 and S4).

### Combinations of antibody responses

To determine if combinations of antibody levels would provide a better predictor of risk than antibody levels to single antigens, the area under the receiver operating characteristic (AUROC) was used to estimate curves for single vs. combination antibody levels for prediction of risk of clinical malaria (Table S5). In each age group, only antibodies for which antibody levels to a single antigen that associated with risk of clinical malaria were included for evaluation. In children < 5 years and 5–14 years, combining antibody levels to different antigens did not improve the prediction of clinical malaria risk compared to single-antigen antibody levels (Table S5). However, in individuals ≥ 15 years of age, the combination of all significant IgG and IgG1 antibody levels showed only a modest increase in predictive value of risk of clinical malaria than single-antigen antibody levels (e.g., AUROC 0.68 for IgG1 to CSP + AMA1 + EBA-175 RIII vs. 0.64 for CSP, Table S5).

## Discussion

In the present study in an area of low and unstable malaria transmission, antibodies to the *P. falciparum* antigens GLURP-R2 and MSP-2 correlated with protection against clinical malaria in individuals < 5 years of age at enrolment, but antibodies to multiple *P. falciparum* antigens were markers solely of an increased risk of malaria in individuals  ≥ 5 years of age. These associations were seen after adjustment for multiple known risk factors for malaria in this area. Combined antibody levels to multiple antigens for clinical malaria risk showed only a modest increase in predictive value for risk of clinical malaria as compared to antibody levels to a single antigen, and the small increase in predictive value was found only in individuals  ≥ 15 years of age. Together, the findings suggest that the immune mechanisms of protection from clinical malaria may change with age in areas of low malaria transmission, and that in individuals  ≥ 5 years of age, antibodies to *P. falciparum* antigens provide information on malaria risk beyond that predicted by standard demographic and geographical factors.

The findings of association of IgG and IgG1 antibodies to GLURP-R2 with protection against clinical malaria in this low transmission area are consistent with the findings of a prior study in this area, with follow-up for 6 years. Together, the findings suggest a consistent association of IgG antibodies with GLURP-R2 with protection against clinical malaria in children < 5 years of age at enrolment in this area.

In contrast, antibodies to numerous vaccine candidate antigens in older children and adults either did not correlate with malaria risk or correlated only with increased risk of malaria. These included antibodies previously associated with protection against clinical malaria in young children in areas of high transmission, including IgG antibodies to CSP [12], AMA-1 [20] and MSP-2 [20], IgG1 antibodies to EBA-175[53], IgG3 antibodies to CSP [21] and MSP-2 [15], and IgM antibodies to EBA-175 [16]. In studies in areas of high malaria transmission that showed an association of antibodies to *P. falciparum* antigens with an increased risk of clinical malaria, the associated was thought to demonstrate either higher exposure risk in the population [21] or lack of antibody levels above a needed threshold [54]. Cases and controls in this study were matched by age and village, and results were adjusted for numerous other risk factors for malaria, so antibody levels to certain antigens appear to be strong markers of intra-village malaria exposure. A previous study in children 1–4 years of age in a high transmission area of Papua New Guinea demonstrated that IgG, IgG1 and/or IgG3 antibodies against MSP-2, AMA-1, EBA-175, EBA-140, and EBA-181 were associated with a higher risk of clinical malaria [54], which was thought to be due to not achieving a threshold level required for protection from clinical malaria. Such an explanation would not apply in the present study, as individuals  ≥ 15 years of age had significantly higher antibody levels than children < 5 years of age, yet antibodies to *P. falciparum* antigens in the older group correlated only with increased risk of clinical malaria.

Prior studies have shown an association between levels of IgM antibodies to different *P. falciparum* blood-stage antigens, including some antigens assessed in the present study, and a reduced risk of clinical malaria [23, 55]. One of these studies also showed a longer persistence of IgM antibodies to *P. falciparum* antigens than has been described for other antigens such as viral antigens [56]. In the present study, the only association of IgM antibodies with risk of clinical malaria was in individuals  ≥ 15 years of age, and in this age group, IgM antibodies to EBA-175 RIII-V and Rh2-2030 were associated with an increased risk of malaria, as was seen for IgG, IgG1, IgG3 and IgA antibodies to other antigens. An earlier study showed a weak association of IgM antibodies with concurrent parasitaemia [57], which was not significant after adjustment for multiple comparisons. In this study, it is unclear if the association was a true association or what mechanism might have caused this if it was a true association. As with IgG and IgA antibody findings in this population, it is hypothesized that the association of increased risk of clinical malaria with IgM antibody levels in older individuals is due to increased malaria exposure in these individuals, though further studies are needed to confirm this hypothesis.

IgG and IgG3 antibodies to MSP-2 were associated with protection against clinical malaria in children < 5 years of age but with an increased risk of clinical malaria in individuals  ≥ 15 years of age. Thus, these antibodies may indicate exposure in older individuals, but do not seem to reflect exposure in children < 5 years of age. Antibodies to MSP-2 have been shown to fix and activate complement, inhibit invasion and promote phagocytosis, supporting their role in potential protection from clinical malaria [32, 56]. The reasons for the differences in association by age in this population require further investigation. One potential explanation is that immune mechanisms most important in protection against clinical malaria change over time, such that IgG antibodies to specific antigens may be important in childhood, but other mechanisms, such a T cell-mediated immunity, may be more important in older children and adults. In addition, other antigen targets, or other antigen conformations or sub-domains, may be important in protection in older children and adults. Future studies should investigate the correlation of a broader range of immune responses to a larger panel of antigens across age with protection against clinical malaria in areas of low and unstable transmission. A major challenge to this evaluation is obtaining and storing samples of peripheral blood mononuclear cells to do T cell testing in a large cohort.

The present study has a number of strengths, including the evaluation of immune correlates of protection in an area of low malaria transmission; the use of a cohort and nested case–control study design to do this evaluation; assessment of multiple antibody subtypes and subclasses to pre-erythrocytic and blood-stage vaccine candidate antigens; and rigorous evaluation and adjustment for risk factors related to malaria exposure. Study limitations include the requirement for a prolonged period of follow-up and therefore a long time between antibody measurement and episode of clinical malaria in some individuals, such that the associations likely do not reflect the antibody levels just prior to the malaria episode. Supporting this contention, most associations or trends with protection from clinical malaria in children < 5 years of age at enrolment were in the first 5 years of follow-up. The lack of direct evaluation of entomological inoculation rate, a standard measure of exposure, due to the very low vector density for most of the year in this site, is an additional limitation.

In summary, the present study shows that in this area of low and unstable malaria transmission, antibodies to the P*. falciparum* antigens GLURP-R2 and MSP-2 correlated with decreased risk of malaria in individuals < 5 years of age, but antibodies to multiple *P. falciparum* antigens were correlated with an increased risk of malaria in individuals  ≥ 5 years of age. Future studies should evaluate whether measures that evaluate specific antibody functions, such as opsonic phagocytosis or complement fixation, or T cell responses to *P. falciparum* antigens are better immune correlates of protection in older individuals in areas of low and unstable malaria transmission.

## Supplementary Information

Supplememtary material 1

## Data Availability

The datasets utilized in the present study are accessible upon reasonable request. Nevertheless, the research was carried out with the approval of the Kenyan Medical Research Institute (KEMRI). The dataset has been included in the supplementary materials. Any additional inquiries or requests should be directed to the principal investigator, Dr. Chandy John.

## Abbreviations

AMA-1: Apical membrane antigen 1
AU: Arbitrary units
AUROC: Area under the receiver operating characteristic curves
CSP: Circumsporozoite protein
EBA-140 RIII-V: Erythrocyte binding antigen 140 region III-V
EBA-175 RII: Erythrocyte binding antigen region II
EBA-175 RIII-V: Erythrocyte binding antigen region III-V
GLURP-R0: Glutamate-rich protein, N-terminal non-repetitive region
GLURP-R2: Glutamate-rich protein, C-terminal repetitive region
IgA: Immunoglobulin A
IgG: Immunoglobulin G
IgG1: Immunoglobulin G1
IgG3: Immunoglobulin G3
IgM: Immunoglobulin M
MFI: Median fluorescence intensity
MSP-1: Merozoite surface protein 1
MSP-2: Merozoite surface protein 2
MSP-3: Merozoite surface protein 3
OR: Odds ratio
Pfs230: *Plasmodium falciparum* Gametocyte surface protein P230
Rh2.2030: Reticulocyte binding proteins homologue 2
Rh4.2: Reticulocyte binding proteins homologue 4
Rh5.1: Reticulocyte binding proteins homologue 5
ROC: Receiver operating characteristic
TRAP: Thrombospondin-related adhesion protein
WHO: World Health Organization
