# Supplementary material for: Antibody correlates of risk of clinical malaria in an area of low and unstable malaria transmission in western Kenya
Source: Malar J. 2025 Mar 4;24:73. doi: 10.1186/s12936-025-05300-1 (PMC11877692; doi:10.1186/s12936-025-05300-1)
Supplement: Supplementary file 1 — Supplememtary material 1 [file 12936_2025_5300_MOESM1_ESM.docx]

**Supplementary Tables and Figures**

**Antibody correlates of risk of clinical malaria in an area of low and unstable malaria transmission in western Kenya**

**Table S1.** Comparison of age between age-matched cases and controls by age category

| Age group | Cases | | Controls | | P value |
| --- | --- | --- | --- | --- | --- |
|  | N | Mean (SD) | N | Mean (SD) |  |
| < 5 years | 80 | 2.64 (1.24) | 240 | 2.84 (1.42) | 0.315 |
| 5-14 years | 103 | 9.32 (2.63) | 309 | 9.34 (2.92) | 0.534 |
| ≥ 15 years | 118 | 32.80 (12.85) | 354 | 32.69 (12.90) | 0.314 |
| All ages | 301 | 16.75 (15.50) | 903 | 16.77 (15.45) | 0.512 |

P values obtained from conditional logistic regression.


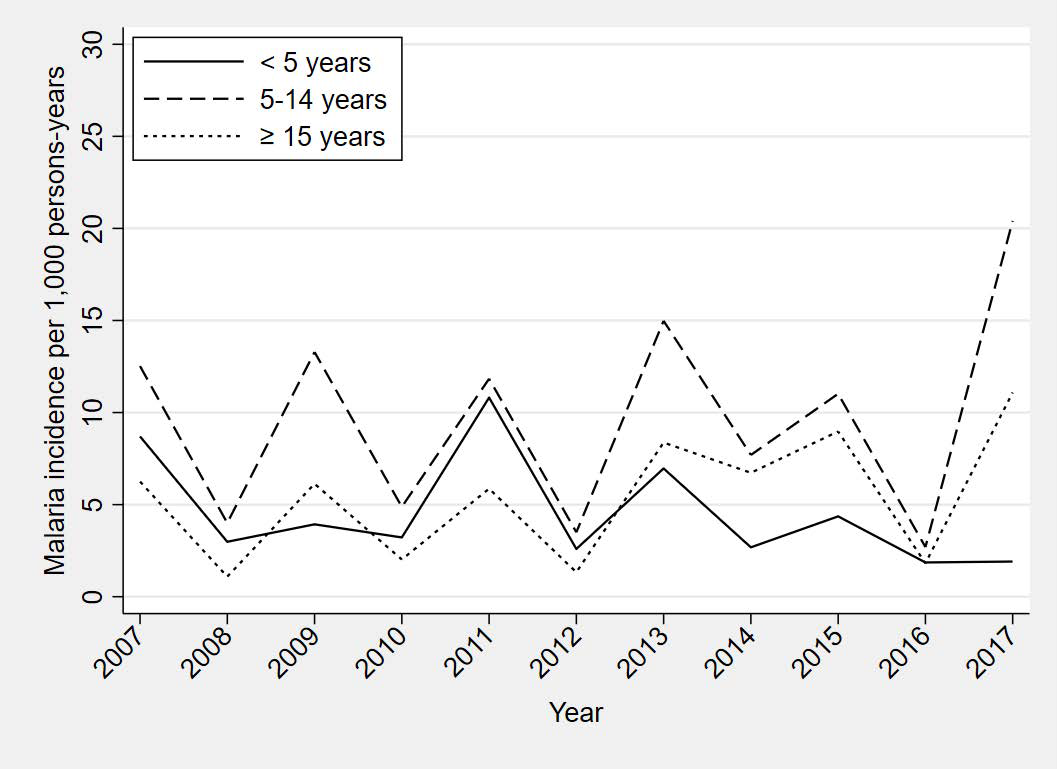
**Figure S1.** Clinical malaria incidence per 1,000 persons in Kipsamoite and Kapsisiywa, Western Kenya, 2007–2017, by age group

**
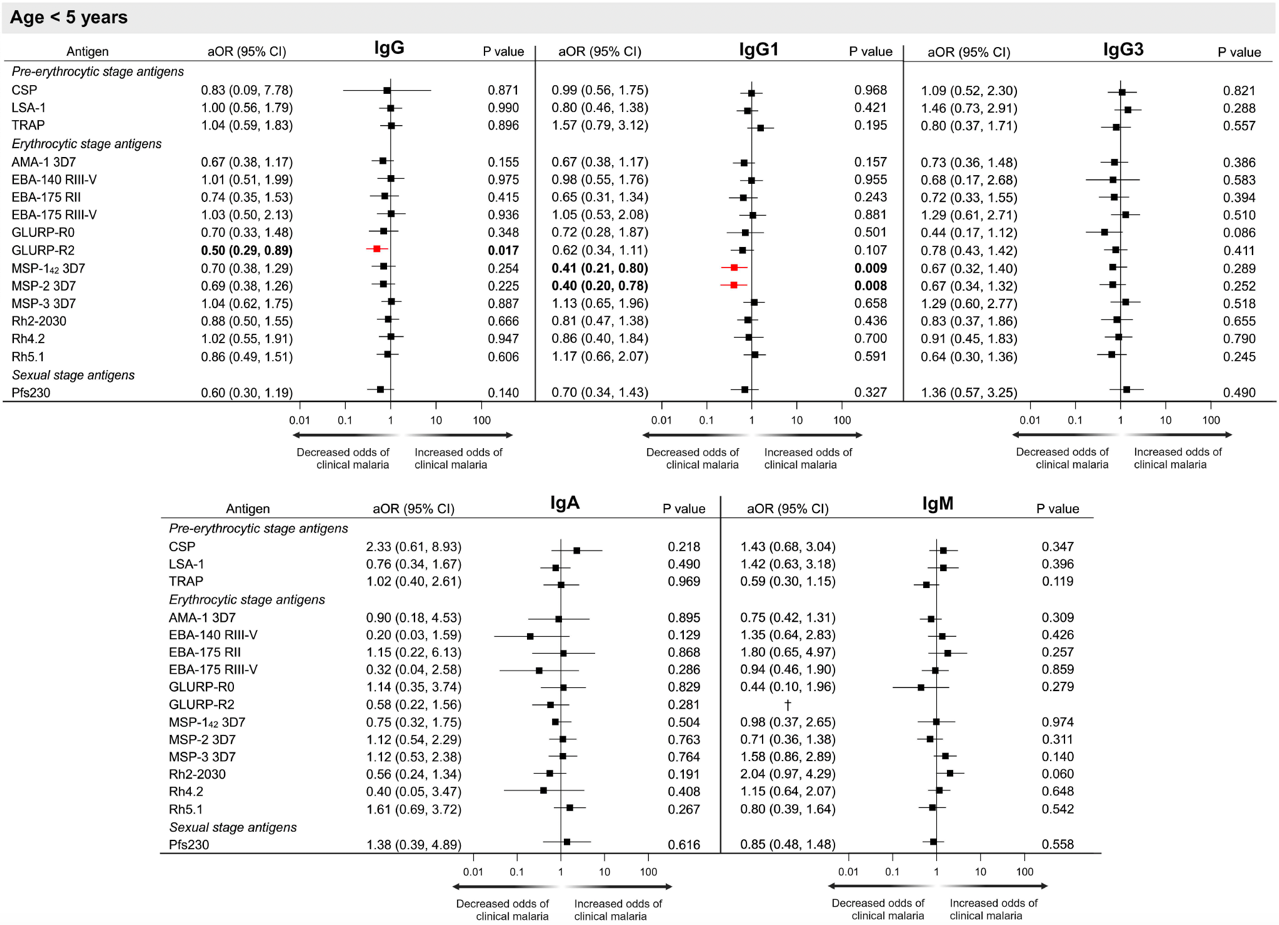
**

**Figure S2.** Forest plots representing the association between IgG, IgG1, IgG3, IgA and IgM dichotomized responses to multiple *Pf* antigens and risk of clinical malaria in children < 5 years. ^a^Adjusted odds ratios (aOR) and p values obtained using conditional logistic regression adjusted for bed net use, household treatment by indoor residual spraying, roof material, distance to nearest forest and elevation. Significant aORs at p < 0.05 are depicted in red.

**
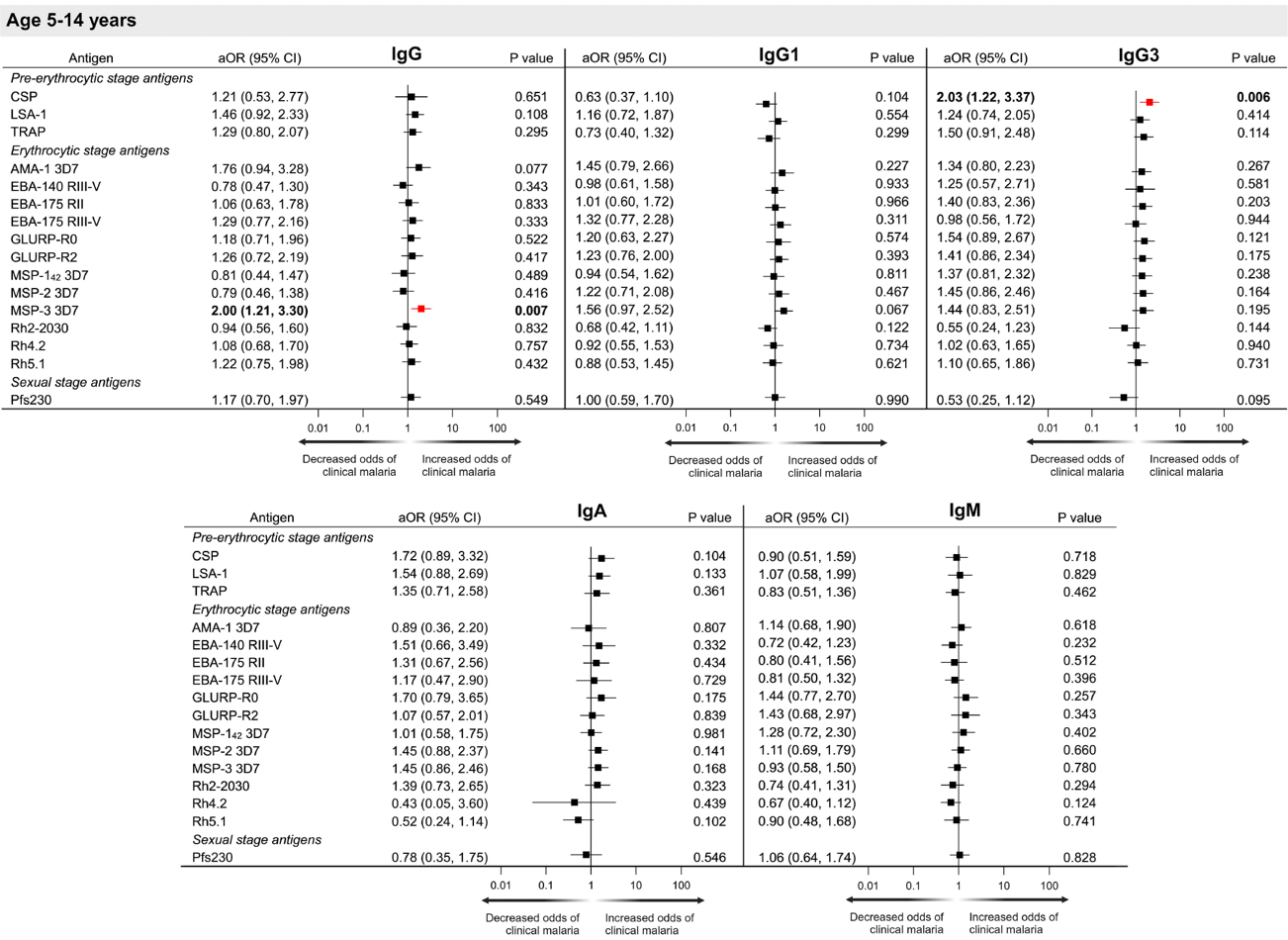
**

**Figure S3.** Forest plots representing the association between IgG, IgG1, IgG3, IgA and IgM dichotomized responses to multiple *Pf* antigens and risk of clinical malaria in children 5-14 years. ^a^Adjusted odds ratios (aOR) and p values obtained using conditional logistic regression adjusted for bed net use, household treatment by indoor residual spraying, roof material, distance to nearest forest and elevation. Significant aORs at p < 0.05 are depicted in red.

**
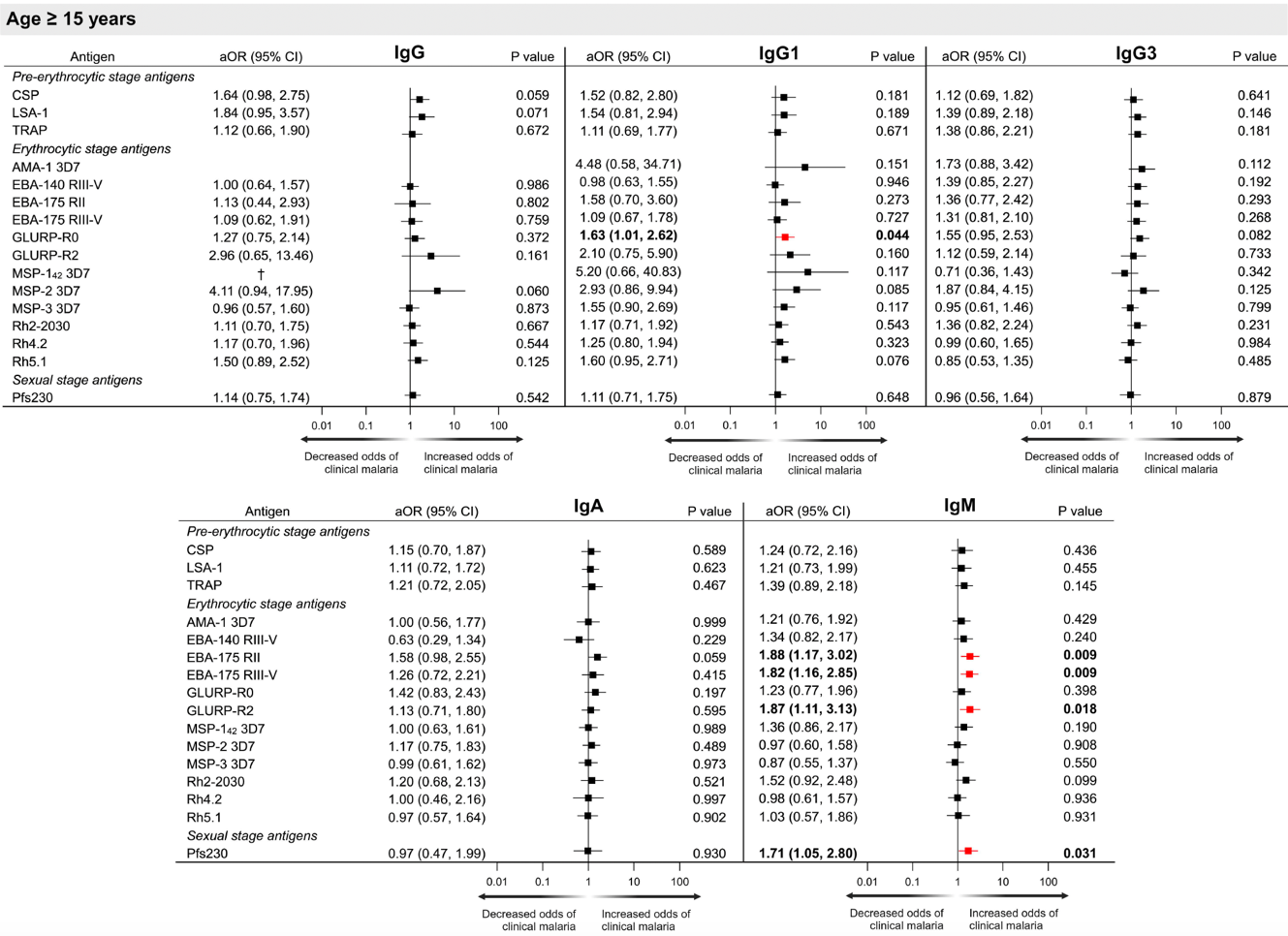
**

**Figure S4.** Forest plots representing the association between IgG, IgG1, IgG3, IgA and IgM dichotomized responses to multiple *Pf* antigens and risk of clinical malaria in individuals ≥ 15 years. ^a^Adjusted odds ratios (aOR) and p values obtained using conditional logistic regression adjusted for bed net use, household treatment by indoor residual spraying, roof material, distance to nearest forest and elevation. Significant aORs at p < 0.05 are depicted in red.

**Table S2**: IgG, IgG1, IgG3, IgA and IgM to multiple *Pf* antigens and association with risk of clinical malaria in years 1-5 vs. 6-10 of follow-up in children < 5 years old

|  | June 2007-June 2012 | | July 2012-May 2017 | |
| --- | --- | --- | --- | --- |
|  | **aOR (95% CI)** | **P value** | **aOR (95% CI)** | **P value** |
| Antigen | **IgG antibody levels** | | | |
| CSP | 0.49 (0.14, 1.71) | 0.261 | 1.48 (0.57, 3.82) | 0.419 |
| LSA-1 | 0.48 (0.16, 1.39) | 0.176 | 1.55 (0.74, 3.27) | 0.246 |
| TRAP | 0.45 (0.08, 2.68) | 0.380 | 1.06 (0.49, 2.28) | 0.882 |
| AMA-1 3D7 | 0.56 (0.26, 1.20) | 0.137 | 0.77 (0.42, 1.39) | 0.381 |
| EBA-140 RIII-V | 0.38 (0.09, 1.61) | 0.189 | 1.39 (0.56, 3.45) | 0.477 |
| EBA-175 RII | 1.08 (0.46, 2.50) | 0.862 | 0.95 (0.48, 1.87) | 0.875 |
| EBA-175 RIII-V | 0.59 (0.09, 3.73) | 0.574 | 2.78 (0.72, 10.75) | 0.139 |
| GLURP-R0, | 0.24 (0.04, 1.43) | 0.116 | 1.27 (0.40, 4.06) | 0.689 |
| GLURP-R2 | 0.32 (0.10, 1.08) | 0.067 | 0.64 (0.33, 1.23) | 0.176 |
| MSP-1_42_ 3D7 | 0.46 (0.19, 1.12) | 0.087 | 0.87 (0.49, 1.57) | 0.651 |
| MSP-2 3D7 | 0.40 (0.14, 1.10) | 0.077 | 0.51 (0.23, 1.09) | 0.082 |
| MSP-3 3D7 | 0.75 (0.20, 2.82) | 0.669 | 1.51 (0.46, 4.96) | 0.492 |
| Rh2-2030 | 0.45 (0.16, 1.29) | 0.138 | 1.05 (0.55, 2.01) | 0.883 |
| Rh4.2 | 0.82 (0.23, 2.89) | 0.756 | 0.84 (0.30, 2.33) | 0.736 |
| Rh5.1 | 1.26 (0.25, 6.38) | 0.777 | 1.32 (0.47, 3.73) | 0.598 |
| Pfs230 | 0.30 (0.07, 1.23) | 0.094 | 0.93 (0.42, 2.10) | 0.866 |
|  | **IgG1 antibody levels** | | | |
| CSP | 0.69 (0.20, 2.44) | 0.566 | 1.27 (0.49, 3.26) | 0.625 |
| LSA-1 | 0.44 (0.12, 1.55) | 0.199 | 1.23 (0.59, 2.58) | 0.577 |
| TRAP | 1.10 (0.15, 8.03) | 0.929 | 0.97 (0.42, 2.21) | 0.940 |
| AMA-1 3D7 | 0.53 (0.25, 1.14) | 0.105 | 0.79 (0.47, 1.34) | 0.382 |
| EBA-140 RIII-V | 0.19 (0.01, 2.63) | 0.218 | 1.24 (0.50, 3.06) | 0.640 |
| EBA-175 RII | 1.04 (0.48, 2.26) | 0.925 | 0.79 (0.37, 1.67) | 0.536 |
| EBA-175 RIII-V | 0.52 (0.00, 105.12) | 0.809 | 0.57 (0.02, 17.02) | 0.745 |
| GLURP-R0, | 0.35 (0.05, 2.62) | 0.305 | 1.31 (0.28, 6.18) | 0.734 |
| GLURP-R2 | 0.33 (0.09, 1.25) | 0.102 | 0.35 (0.14, 0.86) | **0.023** |
| MSP-1_42_ 3D7 | 0.42 (0.16, 1.09) | 0.075 | 0.86 (0.50, 1.47) | 0.572 |
| MSP-2 3D7 | 0.38 (0.10, 1.44) | 0.153 | 0.56 (0.22, 1.44) | 0.230 |
| MSP-3 3D7 | 1.10 (0.15, 8.09) | 0.928 | 2.25 (0.34, 14.76) | 0.396 |
| Rh2-2030 | 0.15 (0.01, 2.16) | 0.162 | 1.34 (0.57, 3.12) | 0.501 |
| Rh4.2 | 0.61 (0.11, 3.47) | 0.577 | 0.67 (0.19, 2.39) | 0.534 |
| Rh5.1 | 0.43 (0.04, 4.98) | 0.502 | 1.19 (0.50, 2.82) | 0.694 |
| Pfs230 | 0.01 (0.00, 2.73) | 0.108 | 1.28 (0.35, 4.72) | 0.710 |
|  | **IgG3 antibody levels** | | | |
| CSP | 1.76 (0.51, 6.08) | 0.373 | 4.02 (0.92, 17.53) | 0.064 |
| LSA-1 | 0.91 (0.23, 3.67) | 0.899 | 1.96 (0.88, 4.34) | 0.097 |
| TRAP | 0.09 (0.00, 170.98) | 0.526 | 0.35 (0.02, 5.26) | 0.447 |
| AMA-1 3D7 | 0.39 (0.01, 11.00) | 0.584 | 0.76 (0.13, 4.34) | 0.754 |
| EBA-140 RIII-V | 0.73 (0.11, 4.98) | 0.751 | 0.70 (0.05, 10.05) | 0.796 |
| EBA-175 RII | 1.16 (0.42, 3.17) | 0.778 | 1.07 (0.32, 3.55) | 0.917 |
| EBA-175 RIII-V | 1.87 (0.33, 10.67) | 0.480 | 2.33 (0.26, 20.61) | 0.446 |
| GLURP-R0, | 0.00 (0.00, 42.49) | 0.212 | 10.90 (0.52, 229.52) | 0.124 |
| GLURP-R2 | 0.26 (0.03, 2.01) | 0.197 | 0.95 (0.49, 1.84) | 0.875 |
| MSP-1_42_ 3D7 | 0.00 (0.00, 36.37) | 0.126 | 0.28 (0.04, 2.04) | 0.209 |
| MSP-2 3D7 | 0.27 (0.06, 1.25) | 0.094 | 0.65 (0.28, 1.53) | 0.324 |
| MSP-3 3D7 | 1.20 (0.11, 13.05) | 0.882 | 0.87 (0.17, 4.45) | 0.863 |
| Rh2-2030 | 0.00 (0.00, 0.08) | **0.031** | 7.91 (0.01, 5789.91) | 0.539 |
| Rh4.2 | 0.24 (0.01, 5.19) | 0.361 | 0.67 (0.05, 8.78) | 0.762 |
| Rh5.1 | 1.00 (0.30, 3.28) | 0.997 | 0.77 (0.23, 2.63) | 0.677 |
| Pfs230 | 0.11 (0.00, 9250.83) | 0.703 | 10.29 (0.27, 393.95) | 0.210 |
|  | **IgA antibody levels** | | | |
| CSP | 0.64 (0.16, 2.66) | 0.542 | 2.59 (0.91, 7.40) | 0.075 |
| LSA-1 | 0.23 (0.03, 2.04) | 0.187 | 1.58 (0.64, 3.90) | 0.325 |
| TRAP | 0.24 (0.03, 2.17) | 0.204 | 1.82 (0.70, 4.75) | 0.220 |
| AMA-1 3D7 | 0.17 (0.01, 2.66) | 0.208 | 0.89 (0.20, 4.07) | 0.884 |
| EBA-140 RIII-V | 0.33 (0.04, 2.93) | 0.321 | 0.85 (0.23, 3.13) | 0.811 |
| EBA-175 RII | 2.43 (0.17, 35.15) | 0.515 | 0.52 (0.05, 5.95) | 0.602 |
| EBA-175 RIII-V | 0.50 (0.03, 9.20) | 0.644 | 0.86 (0.13, 5.43) | 0.868 |
| GLURP-R0, | 0.60 (0.06, 6.23) | 0.673 | 0.94 (0.26, 3.40) | 0.928 |
| GLURP-R2 | 0.07 (0.01, 0.54) | **0.011** | 1.30 (0.62, 2.71) | 0.484 |
| MSP-1_42_ 3D7 | 0.13 (0.01, 2.36) | 0.169 | 0.61 (0.10, 3.75) | 0.597 |
| MSP-2 3D7 | 0.15 (0.03, 0.91) | **0.039** | 3.00 (1.16, 7.76) | **0.023** |
| MSP-3 3D7 | 1.54 (0.13, 19.00) | 0.735 | 1.36 (0.21, 8.92) | 0.752 |
| Rh2-2030 | 0.02 (0.00, 1.32) | 0.068 | 1.34 (0.18, 9.72) | 0.773 |
| Rh4.2 | 0.79 (0.08, 7.42) | 0.839 | 0.96 (0.28, 3.24) | 0.947 |
| Rh5.1 | 1.39 (0.18, 10.93) | 0.752 | 1.94 (0.66, 5.68) | 0.229 |
| Pfs230 | 0.14 (0.00, 5.87) | 0.299 | 1.02 (0.10, 10.39) | 0.988 |
|  | **IgM antibody levels** | | | |
| CSP | 0.86 (0.20, 3.64) | 0.842 | 1.99 (0.70, 5.66) | 0.197 |
| LSA-1 | 2.01 (0.12, 33.55) | 0.627 | 1.16 (0.19, 7.19) | 0.874 |
| TRAP | 0.31 (0.02, 4.77) | 0.398 | 0.65 (0.14, 2.97) | 0.580 |
| AMA-1 3D7 | 1.50 (0.16, 14.03) | 0.724 | 0.71 (0.22, 2.29) | 0.562 |
| EBA-140 RIII-V | 1.23 (0.18, 8.30) | 0.832 | 1.64 (0.42, 6.38) | 0.473 |
| EBA-175 RII | 9.86 (0.50, 194.37) | 0.132 | 1.27 (0.26, 6.32) | 0.766 |
| EBA-175 RIII-V | 1.44 (0.11, 18.96) | 0.782 | 0.92 (0.17, 4.89) | 0.924 |
| GLURP-R0, | 0.63 (0.05, 7.80) | 0.720 | 0.56 (0.10, 3.27) | 0.521 |
| GLURP-R2 | 1.57 (0.17, 14.75) | 0.693 | 0.73 (0.18, 3.05) | 0.669 |
| MSP-1_42_ 3D7 | 0.12 (0.00, 3.69) | 0.228 | 2.85 (0.37, 21.68) | 0.311 |
| MSP-2 3D7 | 1.58 (0.20, 12.27) | 0.661 | 0.37 (0.11, 1.33) | 0.129 |
| MSP-3 3D7 | 5.56 (0.77, 40.07) | 0.089 | 1.22 (0.35, 4.27) | 0.760 |
| Rh2-2030 | 1.40 (0.14, 14.14) | 0.778 | 1.57 (0.30, 8.27) | 0.596 |
| Rh4.2 | 3.46 (0.56, 21.30) | 0.181 | 1.04 (0.34, 3.18) | 0.945 |
| Rh5.1 | 1.03 (0.18, 5.94) | 0.971 | 1.06 (0.30, 3.78) | 0.923 |
| Pfs230 | 1.20 (0.12, 12.42) | 0.881 | 0.95 (0.18, 5.02) | 0.949 |

^a^Adjusted odds ratios (aOR) and p values obtained using conditional logistic regression adjusted for bed net use, household treatment by indoor residual spraying, roof material, distance to nearest forest and elevation. Significant p values (p < 0.05) are bolded.

**Table S3**: IgG, IgG1, IgG3, IgA and IgM to multiple *Pf* antigens and association with risk of clinical malaria in years 1-5 vs. 6-10 of follow-up in children 5-14 years old

|  | June 2007-June 2012 | | July 2012-May 2017 | |
| --- | --- | --- | --- | --- |
|  | **aOR (95% CI)** | **P value** | **aOR (95% CI)** | **P value** |
| Antigen | **IgG antibody levels** | | | |
| CSP | 0.78 (0.35, 1.76) | 0.555 | 1.21 (0.60, 2.43) | 0.600 |
| LSA-1 | 1.02 (0.52, 1.98) | 0.957 | 2.24 (1.20, 4.22) | **0.012** |
| TRAP | 1.19 (0.55, 2.58) | 0.664 | 1.38 (0.72, 2.68) | 0.334 |
| AMA-1 3D7 | 1.42 (0.91, 2.21) | 0.123 | 1.08 (0.70, 1.66) | 0.734 |
| EBA-140 RIII-V | 0.66 (0.27, 1.65) | 0.377 | 0.88 (0.45, 1.75) | 0.722 |
| EBA-175 RII | 1.26 (0.82, 1.95) | 0.298 | 0.89 (0.56, 1.40) | 0.616 |
| EBA-175 RIII-V | 0.82 (0.33, 2.05) | 0.671 | 1.26 (0.61, 2.60) | 0.526 |
| GLURP-R0, | 0.90 (0.43, 1.89) | 0.789 | 1.95 (0.96, 3.94) | 0.064 |
| GLURP-R2 | 0.99 (0.59, 1.67) | 0.985 | 1.27 (0.87, 1.88) | 0.219 |
| MSP-1_42_ 3D7 | 1.27 (0.80, 2.00) | 0.313 | 0.80 (0.52, 1.24) | 0.315 |
| MSP-2 3D7 | 1.01 (0.60, 1.70) | 0.959 | 1.17 (0.74, 1.84) | 0.511 |
| MSP-3 3D7 | 1.02 (0.52, 1.98) | 0.963 | 2.10 (1.00, 4.43) | 0.050 |
| Rh2-2030 | 0.63 (0.24, 1.64) | 0.342 | 0.92 (0.42, 2.01) | 0.837 |
| Rh4.2 | 0.69 (0.32, 1.51) | 0.354 | 1.38 (0.68, 2.80) | 0.371 |
| Rh5.1 | 1.08 (0.44, 2.63) | 0.866 | 0.89 (0.36, 2.19) | 0.804 |
| Pfs230 | 0.69 (0.25, 1.89) | 0.472 | 1.35 (0.65, 2.78) | 0.418 |
|  | **IgG1 antibody levels** | | | |
| CSP | 0.54 (0.24, 1.23) | 0.142 | 1.16 (0.67, 2.01) | 0.597 |
| LSA-1 | 0.76 (0.39, 1.46) | 0.408 | 1.58 (0.95, 2.62) | 0.078 |
| TRAP | 0.94 (0.42, 2.10) | 0.883 | 0.77 (0.33, 1.81) | 0.548 |
| AMA-1 3D7 | 1.27 (0.90, 1.79) | 0.178 | 1.03 (0.73, 1.45) | 0.881 |
| EBA-140 RIII-V | 0.85 (0.36, 2.04) | 0.719 | 0.84 (0.37, 1.90) | 0.668 |
| EBA-175 RII | 1.08 (0.76, 1.54) | 0.653 | 0.89 (0.61, 1.28) | 0.517 |
| EBA-175 RIII-V | 0.57 (0.17, 1.94) | 0.368 | 1.26 (0.59, 2.66) | 0.552 |
| GLURP-R0, | 0.73 (0.34, 1.55) | 0.413 | 1.98 (0.98, 4.01) | 0.058 |
| GLURP-R2 | 0.95 (0.63, 1.44) | 0.818 | 1.27 (0.89, 1.83) | 0.191 |
| MSP-1_42_ 3D7 | 1.23 (0.84, 1.81) | 0.289 | 0.81 (0.57, 1.15) | 0.243 |
| MSP-2 3D7 | 0.96 (0.58, 1.57) | 0.862 | 1.22 (0.79, 1.89) | 0.369 |
| MSP-3 3D7 | 1.32 (0.62, 2.77) | 0.471 | 1.25 (0.51, 3.09) | 0.630 |
| Rh2-2030 | 0.33 (0.04, 2.62) | 0.294 | 0.79 (0.24, 2.61) | 0.701 |
| Rh4.2 | 0.80 (0.35, 1.80) | 0.588 | 0.91 (0.45, 1.84) | 0.795 |
| Rh5.1 | 2.38 (1.13, 5.00) | **0.022** | 0.83 (0.33, 2.09) | 0.692 |
| Pfs230 | 1.01 (0.33, 3.08) | 0.991 | 1.27 (0.58, 2.78) | 0.544 |
|  | **IgG3 antibody levels** | | | |
| CSP | 1.64 (0.87, 3.11) | 0.128 | 1.27 (0.63, 2.58) | 0.506 |
| LSA-1 | 2.27 (1.10, 4.66) | **0.026** | 1.59 (0.80, 3.17) | 0.186 |
| TRAP | 0.66 (0.11, 3.81) | 0.638 | 2.80 (0.76, 10.30) | 0.121 |
| AMA-1 3D7 | 1.37 (0.65, 2.89) | 0.404 | 0.87 (0.46, 1.64) | 0.659 |
| EBA-140 RIII-V | 2.87 (0.86, 9.50) | 0.085 | 1.08 (0.50, 2.33) | 0.836 |
| EBA-175 RII | 1.47 (0.80, 2.72) | 0.217 | 1.06 (0.57, 1.97) | 0.853 |
| EBA-175 RIII-V | 1.27 (0.39, 4.08) | 0.694 | 1.45 (0.61, 3.45) | 0.403 |
| GLURP-R0, | 1.37 (0.46, 4.09) | 0.574 | 4.86 (1.20, 19.65) | **0.027** |
| GLURP-R2 | 1.07 (0.69, 1.67) | 0.751 | 1.26 (0.90, 1.76) | 0.170 |
| MSP-1_42_ 3D7 | 1.05 (0.45, 2.41) | 0.916 | 1.05 (0.48, 2.30) | 0.901 |
| MSP-2 3D7 | 1.20 (0.74, 1.94) | 0.457 | 1.13 (0.76, 1.68) | 0.543 |
| MSP-3 3D7 | 0.85 (0.34, 2.12) | 0.723 | 2.66 (0.96, 7.34) | 0.059 |
| Rh2-2030 | 0.65 (0.03, 15.87) | 0.789 | 1.81 (0.09, 38.04) | 0.703 |
| Rh4.2 | 0.57 (0.18, 1.81) | 0.337 | 1.62 (0.75, 3.50) | 0.223 |
| Rh5.1 | 0.71 (0.26, 1.97) | 0.510 | 0.86 (0.44, 1.68) | 0.660 |
| Pfs230 | 0.01 (0.00, 435.62) | 0.395 | 1.79 (0.24, 13.20) | 0.568 |
|  | **IgA antibody levels** | | | |
| CSP | 1.05 (0.42, 2.65) | 0.917 | 2.58 (1.20, 5.52) | **0.015** |
| LSA-1 | 1.01 (0.33, 3.10) | 0.990 | 3.18 (1.25, 8.07) | **0.015** |
| TRAP | 1.05 (0.38, 2.89) | 0.932 | 3.19 (1.11, 9.15) | **0.031** |
| AMA-1 3D7 | 1.90 (0.50, 7.28) | 0.347 | 1.46 (0.48, 4.48) | 0.505 |
| EBA-140 RIII-V | 1.02 (0.26, 4.05) | 0.976 | 1.44 (0.47, 4.39) | 0.524 |
| EBA-175 RII | 1.43 (0.38, 5.40) | 0.601 | 1.82 (0.64, 5.15) | 0.262 |
| EBA-175 RIII-V | 0.11 (0.01, 1.63) | 0.109 | 2.58 (0.70, 9.52) | 0.155 |
| GLURP-R0, | 1.19 (0.41, 3.46) | 0.751 | 3.80 (1.29, 11.20) | **0.016** |
| GLURP-R2 | 1.49 (0.61, 3.63) | 0.384 | 1.16 (0.62, 2.16) | 0.650 |
| MSP-1_42_ 3D7 | 2.12 (0.56, 8.01) | 0.269 | 1.47 (0.42, 5.15) | 0.549 |
| MSP-2 3D7 | 1.52 (0.65, 3.59) | 0.336 | 1.89 (0.95, 3.78) | 0.071 |
| MSP-3 3D7 | 6.92 (1.03, 46.72) | **0.047** | 4.77 (1.00, 22.79) | 0.051 |
| Rh2-2030 | 0.28 (0.03, 3.18) | 0.307 | 4.55 (0.61, 33.72) | 0.138 |
| Rh4.2 | 1.03 (0.23, 4.61) | 0.972 | 2.51 (0.68, 9.22) | 0.165 |
| Rh5.1 | 0.41 (0.09, 1.80) | 0.236 | 1.74 (0.54, 5.61) | 0.351 |
| Pfs230 | 0.50 (0.08, 3.05) | 0.449 | 4.61 (0.74, 28.79) | 0.102 |
|  | **IgM antibody levels** | | | |
| CSP | 0.34 (0.09, 1.25) | 0.103 | 2.73 (0.79, 9.43) | 0.112 |
| LSA-1 | 0.40 (0.08, 2.07) | 0.274 | 2.00 (0.61, 6.62) | 0.255 |
| TRAP | 0.28 (0.05, 1.53) | 0.141 | 1.47 (0.43, 5.02) | 0.535 |
| AMA-1 3D7 | 0.75 (0.21, 2.72) | 0.666 | 1.53 (0.42, 5.52) | 0.518 |
| EBA-140 RIII-V | 0.45 (0.12, 1.70) | 0.237 | 1.21 (0.35, 4.24) | 0.762 |
| EBA-175 RII | 0.27 (0.05, 1.49) | 0.132 | 0.66 (0.14, 3.17) | 0.601 |
| EBA-175 RIII-V | 0.14 (0.02, 0.83) | **0.030** | 1.62 (0.41, 6.30) | 0.490 |
| GLURP-R0, | 0.81 (0.25, 2.60) | 0.717 | 2.04 (0.74, 5.64) | 0.168 |
| GLURP-R2 | 1.05 (0.32, 3.39) | 0.941 | 1.12 (0.42, 3.01) | 0.815 |
| MSP-1_42_ 3D7 | 0.37 (0.07, 1.90) | 0.235 | 4.22 (1.03, 17.28) | **0.045** |
| MSP-2 3D7 | 0.69 (0.21, 2.32) | 0.548 | 1.63 (0.60, 4.45) | 0.338 |
| MSP-3 3D7 | 0.94 (0.24, 3.61) | 0.928 | 1.73 (0.60, 4.97) | 0.310 |
| Rh2-2030 | 0.12 (0.02, 0.75) | **0.023** | 0.86 (0.22, 3.41) | 0.827 |
| Rh4.2 | 0.27 (0.08, 0.92) | **0.037** | 1.06 (0.34, 3.32) | 0.922 |
| Rh5.1 | 0.80 (0.24, 2.63) | 0.707 | 1.18 (0.35, 4.02) | 0.792 |
| Pfs230 | 0.16 (0.04, 0.69) | **0.014** | 2.13 (0.59, 7.63) | 0.247 |

^a^Adjusted odds ratios (aOR) and p values obtained using conditional logistic regression adjusted for bed net use, household treatment by indoor residual spraying, roof material, distance to nearest forest and elevation. Significant p values (p < 0.05) are bolded.

**Table S4**: IgG, IgG1, IgG3, IgA and IgM to multiple *Pf* antigens and association with risk of clinical malaria in years 1-5 vs. 6-10 of follow-up in individuals ≥ 15 years old.

|  | June 2007-June 2012 | | July 2012-May 2017 | |
| --- | --- | --- | --- | --- |
|  | **aOR (95% CI)** | **P value** | **aOR (95% CI)** | **P value** |
| Antigen | **IgG antibody levels** | | | |
| CSP | 2.15 (0.85, 5.40) | 0.105 | 2.45 (1.35, 4.47) | **0.003** |
| LSA-1 | 1.14 (0.46, 2.85) | 0.773 | 1.16 (0.71, 1.90) | 0.549 |
| TRAP | 1.65 (0.65, 4.19) | 0.292 | 1.35 (0.77, 2.37) | 0.295 |
| AMA-1 3D7 | 2.58 (1.14, 5.84) | **0.023** | 1.54 (0.91, 2.60) | 0.105 |
| EBA-140 RIII-V | 1.16 (0.58, 2.31) | 0.669 | 1.06 (0.72, 1.57) | 0.762 |
| EBA-175 RII | 2.39 (1.21, 4.72) | **0.012** | 1.03 (0.71, 1.49) | 0.868 |
| EBA-175 RIII-V | 2.13 (1.10, 4.13) | **0.024** | 1.14 (0.78, 1.69) | 0.497 |
| GLURP-R0, | 1.21 (0.58, 2.50) | 0.613 | 1.22 (0.80, 1.85) | 0.351 |
| GLURP-R2 | 1.04 (0.51, 2.15) | 0.911 | 1.21 (0.80, 1.81) | 0.363 |
| MSP-1_42_ 3D7 | 2.51 (0.99, 6.34) | 0.052 | 1.17 (0.72, 1.90) | 0.534 |
| MSP-2 3D7 | 1.77 (0.90, 3.48) | 0.097 | 1.33 (0.88, 2.01) | 0.176 |
| MSP-3 3D7 | 1.35 (0.66, 2.77) | 0.408 | 0.83 (0.53, 1.29) | 0.398 |
| Rh2-2030 | 1.12 (0.37, 3.37) | 0.846 | 1.23 (0.67, 2.26) | 0.495 |
| Rh4.2 | 2.21 (0.93, 5.29) | 0.074 | 0.94 (0.58, 1.52) | 0.805 |
| Rh5.1 | 1.77 (0.61, 5.14) | 0.291 | 1.14 (0.63, 2.06) | 0.656 |
| Pfs230 | 1.14 (0.46, 2.85) | 0.772 | 1.21 (0.73, 2.00) | 0.465 |
|  | **IgG1 antibody levels** | | | |
| CSP | 1.58 (0.82, 3.04) | 0.174 | 1.85 (1.17, 2.92) | **0.009** |
| LSA-1 | 0.96 (0.48, 1.89) | 0.897 | 1.04 (0.71, 1.51) | 0.853 |
| TRAP | 1.72 (0.74, 4.00) | 0.204 | 1.10 (0.63, 1.90) | 0.737 |
| AMA-1 3D7 | 2.07 (1.09, 3.92) | **0.026** | 1.60 (1.02, 2.51) | **0.041** |
| EBA-140 RIII-V | 1.07 (0.56, 2.03) | 0.841 | 1.27 (0.88, 1.83) | 0.198 |
| EBA-175 RII | 1.77 (1.09, 2.87) | **0.022** | 1.02 (0.77, 1.34) | 0.906 |
| EBA-175 RIII-V | 1.91 (1.04, 3.50) | **0.037** | 1.28 (0.91, 1.79) | 0.150 |
| GLURP-R0, | 0.87 (0.46, 1.64) | 0.664 | 1.42 (1.01, 2.00) | **0.045** |
| GLURP-R2 | 0.91 (0.51, 1.62) | 0.743 | 1.23 (0.89, 1.70) | 0.208 |
| MSP-1_42_ 3D7 | 2.18 (1.06, 4.48) | **0.034** | 1.08 (0.76, 1.54) | 0.652 |
| MSP-2 3D7 | 1.07 (0.62, 1.87) | 0.797 | 0.99 (0.70, 1.41) | 0.955 |
| MSP-3 3D7 | 1.33 (0.65, 2.73) | 0.434 | 0.95 (0.62, 1.44) | 0.804 |
| Rh2-2030 | 1.37 (0.34, 5.56) | 0.663 | 1.21 (0.67, 2.16) | 0.528 |
| Rh4.2 | 1.45 (0.70, 2.99) | 0.315 | 1.17 (0.77, 1.75) | 0.464 |
| Rh5.1 | 1.66 (0.71, 3.87) | 0.245 | 1.41 (0.89, 2.25) | 0.142 |
| Pfs230 | 1.17 (0.47, 2.90) | 0.732 | 1.27 (0.83, 1.94) | 0.263 |
|  | **IgG3 antibody levels** | | | |
| CSP | 0.98 (0.42, 2.28) | 0.954 | 1.63 (1.11, 2.39) | **0.014** |
| LSA-1 | 1.53 (0.57, 4.11) | 0.396 | 1.56 (1.03, 2.37) | **0.036** |
| TRAP | 1.85 (0.43, 7.99) | 0.409 | 1.85 (0.90, 3.82) | 0.094 |
| AMA-1 3D7 | 1.09 (0.54, 2.22) | 0.807 | 1.35 (0.95, 1.93) | 0.097 |
| EBA-140 RIII-V | 1.10 (0.58, 2.09) | 0.769 | 1.13 (0.82, 1.54) | 0.451 |
| EBA-175 RII | 1.17 (0.64, 2.14) | 0.617 | 0.97 (0.70, 1.32) | 0.827 |
| EBA-175 RIII-V | 1.56 (0.79, 3.11) | 0.204 | 1.30 (0.93, 1.82) | 0.123 |
| GLURP-R0, | 1.55 (0.64, 3.75) | 0.332 | 1.07 (0.68, 1.68) | 0.758 |
| GLURP-R2 | 1.44 (0.88, 2.35) | 0.151 | 1.09 (0.84, 1.42) | 0.500 |
| MSP-1_42_ 3D7 | 1.25 (0.67, 2.36) | 0.481 | 1.10 (0.77, 1.56) | 0.603 |
| MSP-2 3D7 | 1.40 (0.86, 2.27) | 0.172 | 1.29 (0.96, 1.73) | 0.091 |
| MSP-3 3D7 | 0.96 (0.42, 2.23) | 0.932 | 1.05 (0.64, 1.72) | 0.846 |
| Rh2-2030 | 0.29 (0.00, 44.28) | 0.632 | 0.63 (0.07, 5.92) | 0.687 |
| Rh4.2 | 1.76 (0.64, 4.84) | 0.276 | 0.96 (0.61, 1.49) | 0.841 |
| Rh5.1 | 0.69 (0.27, 1.76) | 0.434 | 0.99 (0.56, 1.76) | 0.976 |
| Pfs230 | 0.48 (0.02, 14.81) | 0.672 | 0.46 (0.07, 2.99) | 0.415 |
|  | **IgA antibody levels** | | | |
| CSP | 1.03 (0.40, 2.69) | 0.948 | 1.11 (0.60, 2.05) | 0.746 |
| LSA-1 | 1.46 (0.50, 4.23) | 0.490 | 1.15 (0.60, 2.21) | 0.668 |
| TRAP | 1.03 (0.30, 3.52) | 0.958 | 1.63 (0.75, 3.53) | 0.217 |
| AMA-1 3D7 | 1.82 (0.49, 6.77) | 0.371 | 1.43 (0.61, 3.32) | 0.407 |
| EBA-140 RIII-V | 0.83 (0.16, 4.40) | 0.825 | 1.36 (0.57, 3.25) | 0.483 |
| EBA-175 RII | 3.81 (1.03, 14.11) | **0.045** | 1.51 (0.72, 3.18) | 0.273 |
| EBA-175 RIII-V | 2.35 (0.43, 12.90) | 0.324 | 1.73 (0.64, 4.66) | 0.282 |
| GLURP-R0, | 2.09 (0.62, 6.99) | 0.233 | 2.01 (1.04, 3.91) | **0.038** |
| GLURP-R2 | 0.58 (0.19, 1.79) | 0.347 | 1.52 (0.85, 2.71) | 0.156 |
| MSP-1_42_ 3D7 | 1.73 (0.47, 6.40) | 0.409 | 0.96 (0.39, 2.39) | 0.938 |
| MSP-2 3D7 | 0.72 (0.24, 2.10) | 0.543 | 1.05 (0.54, 2.06) | 0.876 |
| MSP-3 3D7 | 1.16 (0.21, 6.37) | 0.864 | 0.80 (0.26, 2.43) | 0.698 |
| Rh2-2030 | 1.14 (0.07, 19.30) | 0.930 | 3.03 (0.69, 13.18) | 0.140 |
| Rh4.2 | 1.85 (0.41, 8.31) | 0.423 | 0.93 (0.39, 2.22) | 0.865 |
| Rh5.1 | 1.28 (0.35, 4.66) | 0.703 | 0.94 (0.38, 2.31) | 0.885 |
| Pfs230 | 0.33 (0.02, 4.76) | 0.417 | 1.21 (0.38, 3.80) | 0.748 |
|  | **IgM antibody levels** | | | |
| CSP | 0.26 (0.05, 1.45) | 0.125 | 1.72 (0.75, 3.95) | 0.198 |
| LSA-1 | 2.32 (0.65, 8.29) | 0.195 | 0.83 (0.42, 1.65) | 0.594 |
| TRAP | 1.32 (0.31, 5.55) | 0.709 | 1.26 (0.61, 2.60) | 0.534 |
| AMA-1 3D7 | 1.69 (0.55, 5.25) | 0.361 | 1.13 (0.58, 2.22) | 0.720 |
| EBA-140 RIII-V | 1.07 (0.24, 4.82) | 0.927 | 1.54 (0.66, 3.55) | 0.316 |
| EBA-175 RII | 2.44 (0.79, 7.56) | 0.120 | 1.81 (1.03, 3.17) | **0.040** |
| EBA-175 RIII-V | 1.47 (0.50, 4.33) | 0.487 | 1.71 (0.85, 3.47) | 0.134 |
| GLURP-R0, | 1.66 (0.66, 4.21) | 0.283 | 1.17 (0.71, 1.93) | 0.546 |
| GLURP-R2 | 1.59 (0.60, 4.18) | 0.352 | 1.17 (0.68, 2.00) | 0.567 |
| MSP-1_42_ 3D7 | 3.36 (0.78, 14.53) | 0.105 | 1.09 (0.48, 2.45) | 0.840 |
| MSP-2 3D7 | 1.27 (0.40, 4.03) | 0.680 | 0.94 (0.48, 1.85) | 0.858 |
| MSP-3 3D7 | 1.00 (0.28, 3.64) | 0.996 | 0.68 (0.29, 1.61) | 0.385 |
| Rh2-2030 | 4.13 (0.66, 25.90) | 0.130 | 2.34 (0.73, 7.53) | 0.155 |
| Rh4.2 | 0.91 (0.24, 3.38) | 0.882 | 1.27 (0.55, 2.93) | 0.569 |
| Rh5.1 | 1.16 (0.34, 3.96) | 0.810 | 0.66 (0.28, 1.58) | 0.355 |
| Pfs230 | 2.35 (0.41, 13.38) | 0.336 | 0.96 (0.38, 2.46) | 0.935 |

^a^Adjusted odds ratios (aOR) and p values obtained using conditional logistic regression adjusted for bed net use, household treatment by indoor residual spraying, roof material, distance to nearest forest and elevation. Significant p values (p < 0.05) are bolded.

**Table S5.** Comparison of areas under the receiver operating characteristic curves for combinations of antibody levels associated with the development of clinical malaria by age group and antibody subclass

| **Ages < 5 years** | **AUROC (95% CI)** | **P value** |
| --- | --- | --- |
| **IgG** |  |  |
| GLURP-R2 + MSP-2 vs. GLURP-R2 | 0.67 (0.60, 0.74) vs. 0.64 (0.58, 0.71) | 0.145 |
| GLURP-R2 + MSP-2 vs. MSP-2 | 0.67 (0.60, 0.74) vs. 0.66 (0.59, 0.73) | 0.504 |
| **Ages 5-14 years** | **AUROC (95% CI)** | **P value** |
| **IgG3** |  |  |
| LSA-1 + GLURP-R0 vs. LSA-1 | 0.66 (0.60, 0.72) vs. 0.65 (0.59, 0.71) | 0.415 |
| LSA-1 + GLURP-R0 vs. GLURP-R0 | 0.66 (0.60, 0.72) vs. 0.64 (0.59, 0.70) | 0.289 |
| **IgA** |  |  |
| CSP + GLURP-R0 + MSP-2 + MSP-3 vs. CSP | 0.67 (0.61, 0.73) vs. 0.64 (0.58, 0.70) | 0.134 |
| CSP + GLURP-R0 + MSP-2 + MSP-3 vs. GLURP-R0 | 0.67 (0.61, 0.73) vs. 0.64 (0.58, 0.70) | 0.179 |
| CSP + GLURP-R0 + MSP-2 + MSP-3 vs. MSP-2 | 0.67 (0.61, 0.73) vs. 0.65 (0.59, 0.71) | 0.312 |
| CSP + GLURP-R0 + MSP-2 + MSP-3 vs. MSP-3 | 0.67 (0.61, 0.73) vs. 0.66 (0.60, 0.72) | 0.662 |
| **Ages ≥ 15 years** | **AUROC (95% CI)** | **P value** |
| **IgG** |  |  |
| CSP + AMA-1 + MSP-2 vs. CSP | 0.67 (0.61, 0.73) vs. 0.65 (0.59, 0.71) | 0.118 |
| CSP + AMA-1 + MSP-2 vs. AMA-1 | 0.67 (0.61, 0.73) vs. 0.63 (0.57, 0.69) | 0.055 |
| CSP + AMA-1 + MSP-2 vs. MSP-2 | 0.67 (0.61, 0.73) vs. 0.61 (0.55, 0.67) | **0.013** |
| **IgG1** |  |  |
| CSP + AMA-1 + EBA-175 RIII vs. CSP | 0.68 (0.62, 0.73) vs. 0.64 (0.58, 0.69) | **0.031** |
| CSP + AMA-1 + EBA-175 RIII vs. AMA-1 | 0.68 (0.62, 0.73) vs. 0.64 (0.58, 0.70) | **0.030** |
| CSP + AMA-1 + EBA-175 RIII vs. EBA-175 RIII | 0.68 (0.62, 0.73) vs. 0.62 (0.56, 0.68) | **0.008** |
| **IgG3** |  |  |
| CSP + LSA-1 + MSP-2 vs. CSP | 0.64 (0.58, 0.70) vs. 0.62 (0.56, 0.68) | 0.275 |
| CSP + LSA -1 + MSP-2 vs. LSA -1 | 0.64 (0.58, 0.70) vs. 0.61 (0.55, 0.67) | 0.146 |
| CSP + LSA -1 + MSP-2 vs. MSP-2 | 0.64 (0.58, 0.70) vs. 0.61 (0.55, 0.67) | 0.146 |
| **IgM** |  |  |
| EBA-175 RII + Rh2-2030 vs. EBA-175 RII | 0.64 (0.58, 0.70) vs. 0.62 (0.56, 0.69) | 0.259 |
| EBA-175 RII + Rh2-2030 vs. Rh2-2030 | 0.64 (0.58, 0.70) vs. 0.61 (0.56, 0.67) | 0.230 |

Areas under the receiving operating characteristic (AUROC) curves obtained from predicted probabilities of clinical malaria following conditional logistic regression. Antibody levels were log transformed, and regression model adjusted for adjusted for bed net use, household treatment by indoor residual spraying, roof material, distance to nearest forest and elevation. Models incorporating all significant antibody levels were compared to those including single antibody levels and assessed for equality. Significant p values (p < 0.05) are bolded.
